# Is routine cardiac MRI justifiable in patients with non-ischemic cardiomyopathy?

**DOI:** 10.1186/1532-429X-11-S1-P202

**Published:** 2009-01-28

**Authors:** Gorgi Kozeski, M Rizwan Khalid, Fatima R Khalid, Hormoz Kianfar, Anita Kozeska, Tarek Mousa, Ola Akinboboye

**Affiliations:** 1 New York Hospital Medical Center of Queens, Flushing, NY USA; 2 Emory University, Atlanta, GA USA

**Keywords:** Cardiomyopathy, Left Ventricular Ejection Fraction, Cardiac Magnetic Resonance, Late Gadolinium Enhancement, Cardiac Magnetic Resonance Image

## Introduction

According to the recently published appropriateness criteria, evaluation of left ventricular (LV) function in patients with heart failure is an uncertain indication for Cardiac Magnetic Resonance Imaging (CMR). However, in addition to assessment of LV function, other ancillary findings on a cardiac MRI study may alter management decisions in patients with heart failure. An example is the extent of delayed enhancement, which has been shown to be a major prognostic indicator in patients with non-ischemic cardiomyopathy (NICM).

## Purpose

We hypothesize that a CMR study often provides information that alters the course of management in patients with non-ischemic cardiomyopathy.

## Methods

We conducted a retrospective analysis of 112 consecutive patients who underwent CMR with or without gadolinium at our center from June 2007 to April 2008. Forty-three patients were diagnosed as having a cardiomyopathy. Cardiomyopathy was defined as a left ventricular ejection fraction (LVEF) of less than 50%. Twenty-four of these patients were diagnosed with NICM. All patients with NICM had previous negative ischemia evaluation.

### Cardiac MRI study

After performing standard localizer images, ECG-gated breath-hold segmented k-space SSFP images were obtained in standard projections. T2-weighted and delayed enhancement images were also performed.

## Results

A total of 24 patient studies were examined, including 10 males and 14 females with a mean age of 52 ± 16 years. Average LVEF was found to be 33 ± 13 (%). Of these, 7 (29%) had significant management changes and therapeutic interventions guided by the CMR results. Six patients had evidence of late gadolinium enhancement (LGE), and in one patient gadolinium was not given due to renal insufficiency. In this patient, T2-weighted images revealed evidence of increased signal intensity suggestive of myocardial inflammation. The results of CMR in these patients and specific management changes are summarized in Table 1.

**Table 1:** Description of seven patients with NICM in which CMR revealed a specific diagnosis and changed management course

Patients	CMR diagnosis	Evidence of LGE	Management
Patient 1	Newly diagnosed constrictive pericarditis	Present	Surgical evaluation for pericardial stripping
Patient 2	Newly diagnosed cardiac sarcoidosis	Not performed due to renal insufficiency (T2 weighted imaging shows evidence of increased signal intensity suggestive of myocardial inflammation)	IV Steroids Implantable cardioverter defibrillator (ICD) implantation
Patient 3	Newly diagnosed cardiac sarcoidosis	Present	IV steroids ICD implantation
Patient 4	Newly diagnosed non-compaction cardiomyopathy	Present	Electrophysiologic evaluation and ICD implantation
Patient 5	Newly diagnosed left ventricular mass	Present	Urgent surgical evaluation
Patient 6	Newly diagnosed cardiac amyloid (AL-type by kidney biopsy) Newly diagnosed large bilateral pleural effusions with collapsed left lung	Present	Urgent thoracocentesis and further evaluation of cardiac amyloidosis
Patient 7	Myocarditis with large right pleural effusion	Present	Aggressive diuresis and management of myocarditis

## Conclusion

Cardiac Magnetic Resonance Imaging is an invaluable tool in the management of patients with NICM. In our case review, approximately one-third of the patients had significant management changes guided by the CMR results, and almost all patients had presence of late gadolinium enhancement.

